# Epidemiology of Otitis Media with Spontaneous Perforation of the Tympanic Membrane in Young Children and Association with Bacterial Nasopharyngeal Carriage, Recurrences and Pneumococcal Vaccination in Catalonia, Spain - The Prospective HERMES Study

**DOI:** 10.1371/journal.pone.0170316

**Published:** 2017-02-01

**Authors:** Robert Cilveti, Montserrat Olmo, Josefa Pérez-Jove, Juan-José Picazo, Josep-Lluis Arimany, Emiliano Mora, Tomás M. Pérez-Porcuna, Ignacio Aguilar, Aurora Alonso, Francesc Molina, María del Amo, Cristina Mendez, on behalf of the HERMES Study Group

**Affiliations:** 1 Department of Paediatrics, H. Universitari Mútua Terrassa, Barcelona, Spain; 2 Catlab, Viladecavalls, Barcelona, Spain; 3 School of Medicine, Universidad Complutense, Madrid, Spain; 4 H. General de Cataluña, Barcelona, Spain; 5 CAP Valldoreix, Barcelona, Spain; 6 CAP Turó de Can Mates, San Cugat del Vallés, Barcelona, Spain; 7 CAP Rambla, Barcelona, Spain; 8 CAP Terrassa Sud, Barcelona, Spain; 9 Medical Department, Pfizer, Madrid, Spain; Instituto Butantan, BRAZIL

## Abstract

The Epidemiology of otitis media with spontaneous perforation of the tympanic membrane and associated nasopharyngeal carriage of bacterial otopathogens was analysed in a county in Catalonia (Spain) with pneumococcal conjugate vaccines (PCVs) not included in the immunization programme at study time. A prospective, multicentre study was performed in 10 primary care centres and 2 hospitals (June 2011-June 2014), including all otherwise healthy children ≥2 months ≤8 years with otitis media presenting spontaneous tympanic perforation within 48h. Up to 521 otitis episodes in 487 children were included, showing by culture/PCR in middle ear fluid (MEF): *Haemophilus influenzae* [24.2%], both *Streptococcus pneumoniae* and *H*. *influenzae* [24.0%], *S*. *pneumoniae* [15.9%], *Streptococcus pyogenes* [13.6%], and *Staphylococcus aureus* [6.7%]. Culture-negative/PCR-positive otitis accounted for 31.3% (*S*. *pneumoniae*), 30.2% (*H*. *influenzae*) and 89.6% (mixed *S*. *pneumoniae/H*. *influenzae* infections). Overall, incidence decreased over the 3-year study period, with significant decreases in otitis by *S*. *pneumoniae* and by *H*. *influenzae*, but no decreases for mixed *S*. *pneumoniae/H*. *influenzae* infections. Concordance between species in nasopharynx and MEF was found in 58.3% of cases, with maximal rates for *S*. *pyogenes* (71.8%), and with identical pneumococcal serotype in 40.5% of cases. Most patients (66.6%) had past episodes. PCV13 serotypes were significantly more frequent in first episodes, in otitis by *S*. *pneumoniae* as single agent, and among MEF than nasopharyngeal isolates. All non-PCV13 serotypes separately accounted for <5% in MEF. Up to 73.9% children had received ≥1 dose of PCV, with lower carriage of PCV13 serotypes than among non-vaccinated children. Pooling pneumococcal isolates from MEF and nasopharynx, 30% were multidrug resistant, primarily belonging to serotypes 19A [29.8%], 24A [14.3%], 19F [8.3%] and 15A [6.0%]. Our results suggest that increasing PCV13 vaccination would further reduce transmission of PCV13 serotypes with special benefits for youngest children (with none or uncompleted vaccine schedules), preventing first otitis episodes and subsequent recurrences.

## Introduction

Otitis media remains a major public health problem in early childhood worldwide, with the highest incidence between 6 and 24 months of age. By their third birthday, 80% of children will have experienced at least one episode (about half ≥3 episodes) [1] and 40% of children will have 6 or more recurrences by the age of 7 years [2].

The nasopharynx is a complex microbiome where hundreds of bacterial species and viruses interact with each other, and constitutes the ecologic reservoir of bacteria causing acute otitis media. Only few colonising bacterial species (the so-called otopathogens) are implicated in otitis media: *Streptococcus pneumoniae*, non-typeable *Haemophilus influenzae* (by far the two most common), *Streptococcus pyogenes*, *Staphylococcus aureus* and *Moraxella catarrhalis*. Progression from commensal to pathogenic state is facilitated by viral upper respiratory tract infections in 95% of cases caused by *S*. *pneumoniae* and non-typeable *H*. *influenzae* [3,4]. However, for etiological diagnosis, the positive predictive value of nasopharyngeal cultures is poor because of the high number of bacteria present [5], with detection of bacterial species in middle ear fluid (MEF) remaining the gold standard method [6].

Because nasopharyngeal colonization is the first step in otitis media, understanding the impact of therapeutic/preventive measures on nasopharyngeal microbiota is essential [7,8]. Changes in nasopharyngeal carriage of otopathogens have been primarily assessed after the introduction of pneumococcal conjugate vaccines (PCVs) [7,9–15], with marked reductions in carriage of PCV13 serotypes and increases in non-vaccine serotypes after PCV7/13 introduction [10,12,14]. Importantly, a high PCV coverage results in herd protection against pneumococcal carriage, and this may be important for children too young to reach ≥2 doses of PCVs [15]. Immunization may have also created ecological niches favouring colonization by other otopathogens such as non-typeable *H*. *influenzae* and *S*. *aureus*, with postulated subsequent shifts in otitis by these organisms [7,9–16]. In this regard, after PCV7 introduction an increase in the incidence of otitis media by non-vaccine serotypes along with non-pneumococcal cases was reported, resulting in a modest efficacy against all-cause otitis media [15].

In contrast with most European countries, in Spain PCVs (PCV7 since October 2001, replaced by PCV13 on June 2010) were not initially included in regional paediatric immunization programmes for healthy children (except in two regions, Madrid and Galicia), and were only available in the private market. Although implementation in the different regions is due by December 2016, at the study time PCV13 was not included in the paediatric immunization programme in Catalonia.

The aim of this study was to describe bacterial aetiology of otitis media with spontaneous perforation of the tympanic membrane and associated nasopharyngeal carriage of bacterial otopathogens in children younger than 8 years old, in a well-defined county in Catalonia (Spain) when PCVs were not included in the regional immunization programme.

## Materials and Methods

### Study design and data collection

A 3-year epidemiological, prospective, multicentre study was performed in 10 primary care centres located in a well-defined county (Vallés Occidental) in Catalonia, Spain, and two nearby hospitals (Hospital Universitari Mútua Terrassa and Hospital General de Cataluña) from June 2011 to June 2014. All otherwise healthy children aged ≥2 months ≤8 years old attending the study centres with a diagnosis of otitis media presenting spontaneous perforation of the tympanic membrane within the previous 48h (OM), were included after obtaining the written informed consent of parents/guardians. Children could be included on more than one occasion (and were considered as new cases) if they presented new episodes of OM during the study period (defining new episodes as OM caused by a microorganism different from the one isolated >15 days earlier). The study protocol was approved by the Ethical Committees of Hospital Universitari Mútua Terrassa (Terrassa, Catalonia, Spain) and the Hospital General de Cataluña (Sant Cugat del Vallés, Catalonia, Spain).

Children were treated according to the criteria of attending physicians. MEF samples were obtained from spontaneously draining ears by using sterile swabs (eSwab, COPAN, Brescia, Italy) to collect a sample of the discharge. Nasopharyngeal samples were obtained by nasopharyngeal lavage with at least 1.5 ml of saline solution or by gently passing and quickly rotating a sterile cotton-tipped swab through a nostril and into the nasopharynx. As per the daily routine, samples were appropriately packaged, stored at 4°C and sent daily from primary care centres/hospitals to the Centre d’Analítiques Clíniques Catlab (Viladecavalls, Catalonia, Spain) for microbiological culture.

For each episode, demographic and clinical data were recorded including age, gender, highest recorded body temperature, presence of unilateral or bilateral otitis, previous history of otitis media, previous history of ear, nose and throat (ENT) surgery, antibiotic treatment (previous 30 days), and history of previous pneumococcal vaccination.

### Microbiological procedures

Conventional microbiological culture of samples from the ear and nasopharynx (Columbia CNA + 5% sheep blood, Chocolate PolyViteX TM agar, MacConkey agar, Biomerieux, Marcy—Etoile, France) was performed. The identification of the microorganism was carried out by Matrix-assisted laser desorption/ionization (MALDI), Biomerueux. Isolates identified at the Centre d’Analítiques Clíniques Catlab were appropriately labelled, frozen at -80°C, and sent monthly to the Microbiology department of Hospital Clínico Universitario San Carlos (Madrid, Spain) for re-identification and analysis. Pneumococcal isolates were serotyped using latex (Pneumotest-latex, Statens Serum Institut, Copenhagen, Denmark) and Quellung reaction using pneumococcal antisera (Statens Serum Institut, Copenhagen, Denmark). Culture negative samples were also sent to the Hospital Clínico Universitario San Carlos for PCR detection of *S*. *pneumoniae* and *H*. *influenzae* [17,18]. Pneumococci confirmed by PCR were serotyped by real-time PCR assay as previously described [19], detecting serotypes 1, 3, 4, 5, 6, 7F, 14, 19A and 19F.

Antibiotic susceptibility was determined by microdilution following CLSI recommendations [20]. The production of β-lactamase by *H*. *influenzae* isolates was determined using nitrocefin disks (Becton Dickinson, Cockeysville, MD, USA). The macrolide resistance phenotypes in *S*. *pneumoniae* were determined by the double disc diffusion method with erythromycin (15μg) and clindamycin (2μg) discs on Mueller-Hinton agar supplemented with 5% sheep blood. The plates were incubated overnight in 5% CO_2_ atmosphere at 35°C. The *erm* and *mef* genes were detected by PCR [21], with a subsequent PCR to differentiate between *mef* genes [22].

Current CLSI breakpoints [23] were considered for susceptibility interpretation, choosing non-meningitis breakpoints and breakpoints for oral compounds, when applicable, due to the clinical entity analysed in the present study. Isolates with intermediate or high-level resistance were defined as non-susceptible. Multidrug resistance was considered as non-susceptibility to three or more resistance markers for antibiotic groups: penicillin for β-lactams, erythromycin for macrolides/azalides, clindamycin for lincosamides and tetracycline.

### Statistical analysis

Annual population data on children ≥2 months ≤8 years old and estimated person-years data for this age group were obtained from Padró municipal d'habitants, Institut d'Estadística de Catalunya (Idescat), Catalonia, Spain (www.idescat.cat).

Incidence rates (IR) per 100,000 inhabitants of the study age group were calculated globally for all study periods and for each period separately (period I: 14 June 2011–30 June 2012, period II: 1 July 2012–30 June 2013, period III: 1 July 2013–30 June 2014), for all OM and for each specific etiological OM group. Incidence rates ratios (IRRs), with their respective 95% confidence intervals, were calculated. Comparisons were performed with the EPIDAT version 3.1 (Conselleria de Sanidade, Xunta de Galicia, Spain, and Pan-American Health Organization, Washington D.C., USA).

Comparisons between proportions were performed by the χ2 test, the Fisher’s exact test and the Likelihood ratio test, as necessary. For quantitative variables, the Student’s t-test or the ANOVA test were used, and when data did not show normality in the Kolmogorov-Smirnoff test, the Kruskal-Wallis and Mann-Whitney tests were used instead.

Several stepwise logistic regression multivariate analyses were conducted in order to determine factors associated with: 1) first episode of otitis, 2) otitis by *S*. *pneumoniae* (as single agent or mixed infections), 3) first episode of otitis by *S*. *pneumoniae* (as single agent or mixed infections), 4) otitis caused by PCV13 serotypes (as single agent or mixed infections), 5) otitis by *H*. *influenzae* (as single agent or mixed infections), and 6) mixed infection (otitis by *S*. *pneumoniae* plus *H*. *influenzae*). All variables showing differences in univariate analyses (p <0.1) were considered for inclusion in the models. Interactions and linear dependence between independent variables were previously controlled. Models showing the maximum parsimony (the lowest number of variables with no significant reduction in the value of the determination coefficient) and the highest R^2^ were considered. Statistical analyses were performed using the SPSS v 19.0 program (SPSS Inc, Chicago, IL, USA).

## Results

### Study population and aetiology

A total of 521 episodes of OM in 487 children were included in the 3-year study period: 230 in period I, 167 in period II and 124 in period III. Twenty-seven participating children had repeated episodes within the whole study period: one child had 5 episodes, one had 4, two had 3 episodes, and 23 children had 2 episodes. Overall, *H*. *influenzae* was identified in 126 (24.2%) MEF samples, both *S*. *pneumoniae* and *H*. *influenzae* in 125 (24.0%), *S*. *pneumoniae* in 83 (15.9%), *Streptococcus pyogenes* in 71 (13.6%), *Staphylococcus aureus* in 35 (6.7%), other microorganisms (20 Gram-negative bacteria, 5 Gram-positive bacteria and 15 fungi) in 40 (7.7%), and in 41 (7.9%) samples no microorganism could be cultured/PCR identified. Approximately one-third of OM by *S*. *pneumoniae* (26/83; 31.3%) and of those by *H*. *influenzae* (38/126; 30.2%) as single agents were diagnosed by PCR (culture-negative samples). The percentage increased to 89.6% (112/125) for mixed infections by *S*. *pneumoniae* and *H*. *influenzae*. The distribution of cases diagnosed by PCR among all *S*. *pneumoniae* and *H*. *influenzae* cases was similar for children who had taken antibiotics within the previous 30 days and those who had not.

### Epidemiological/clinical characteristics and associations with etiological agents

Table 1 shows IRs and IRRs by etiological agents in the different study periods. Overall, IR of OM significantly decreased over the 3-year study period, with significant decreases in OM by *S*. *pneumoniae* (p<0.001 for IRR 2012-13/2011-12 and IRR 2013-14/2011-12), by *H*. *influenzae* (p<0.001 for IRR 2013-14/2011-12 and IRR 2013-14/2012-13), and by *S*. *pyogenes* (p<0.05 for IRR 2013-14/2011-12).

**Table 1:** Incidence rates (no. cases/100,000 inhabitants aged ≥2 months–<8 years old in the Vallés Occidental, Catalonia, Spain, and incidence rate ratios (IRR) of OM caused by the indicated microorganisms.

	All periods	2011–12	2012–13	2013–14	IRR 2012–13 / 2011–12	IRR 2013–14 / 2011–12	IRR 2013–14 / 2012–13
**Population**	127,398	42,431	42,793	42,174			
***S*. *pneumoniae***	65.15	113.12	46.74	35.57	0.41 (0.25–0.70) p<0.001	0.31 (0.18–0.56) p<0.001	0.76 (0.39–1.49) p = 0.527
***H*. *influenzae***	98.90	136.69	114.50	45.05	0.84 (0.57–1.23) p = 0.414	0.33 (0.20–0.55) p<0.001	0.39 (0.23–0.67) p<0.001
***S*. *pneumoniae + H*. *influenzae***	98.12	91.91	102.82	99.59	1.12 (0.73–1.72) p = 0.689	1.08 (0.70–1.68) p = 0.803	0.97 (0.63–1.48) p = 0.968
***S*. *pyogenes***	55.73	75.42	51.41	40.31	0.68 (0.40–1.17) p = 0.209	0.53 (0.30–0.96) p = 0.048	0.78 (0.42–1.48) p = 0.552
***S*. *aureus***	27.47	28.28	21.03	33.20	0.74 (0.31–1.76) p = 0.649	1.17 (0.54–2.54) p = 0.832	1.58 (0.68–3.65) p = 0.385
**Other**	31.40	51.85	18.69	23.71	0.36 (0.16–0.81) p = 0.017	0.46 (0.22–0.97) p = 0.054	1.27 (0.50–3.21) p = 1.000
**Negative culture/PCR**	32.18	44.78	35.05	16.60	0.78 (0.40–1.54) p = 0.590	0.37 (0.16–0.88) p = 0.032	0.47 (0.19–1.16) p = 0.145
**Total**	408.95	542.06	390.25	294.02	0.72 (0.59–0.88) p = 0.001	0.54 (0.44–0.67) p<0.001	0.75 (0.60–0.95) p = 0.019

Over the 3-year period, cases followed a seasonal pattern with peaks in late winter and troughs in middle summer. This seasonality was more evident in cases caused by *H*. *influenzae* with or without *S*. *pneumoniae*. Up to 95.4% of episodes occurred in children who presented risk factors, previous episodes of OM, common cold in the previous 15 days and day care attendance being the most frequent. Table 2 shows demographic data distributed by species identified in MEF. Age <24 months was found to be positively associated with otitis by *S*. *pneumoniae* (OR = 2.11; 95%CI = 1.19, 3.73; p = 0.010) in the multivariate model performed (R^2^ = 0.019, p = 0.024) (see S1 Table for univariate analysis). Antibiotic intake in the previous 30 days was significantly (p<0.01) higher in patients with episodes involving *H*. *influenzae* or *S*. *aureus*.

**Table 2:** Demographic data of children included in the study distributed by isolates from middle ear fluid.

	SP	Hi	SP + Hi	*S*. *pyogenes*	*S*. *aureus*	Other	Negative culture/PCR	Total
n	83	126	125	71	35	40	41	521
Age, median (P_25_, P_75_)^a^	22.0	21.0	21.0	30.0	43.0	29.0	34.0	24.0
	(11.0, 38.0)	(15.0, 32.0)	(14.0, 38.0)	(16.0, 55.0)	(20.0, 60.0)	(15.0, 55.0)	(15.0, 63.0)	(14.0, 44.0)
<12m	23 (27.7)	21 (16.7)	23 (18.4)	8 (11.3)	5 (14.3)	6 (15.0)	8 (19.5)	94 (18.0)
≥12 –<24m	22 (26.5)	52 (41.3)	48 (38.4)	18 (25.4)	6 (17.1)	10 (25.0)	9 (22.0)	165 (31.7)
≥24–<60m	29 (34.9)	45 (35.7)	43 (34.4)	32 (45.1)	15 (42.9)	12 (30.0)	14 (34.1)	190 (36.5)
≥60m	9 (10.8)	8 (6.3)	11 (8.8)	13 (18.3)	9 (25.7)	12 (30.0)	10 (24.4)	72 (13.8)
Males	52 (62.7)	75 (59.5)	70 (56.0)	45 (63.4)	13 (37.1)	24 (60.0)	25 (61.0)	304 (58.3)
Common cold (previous 15 days)	60 (72.3)	78 (61.9)	86 (68.8)	48 (67.6)	24 (68.6)	21 (52.5)	21 (51.2)	338 (64.9)
Attending day care centres	48 (57.8)	86 (68.3)	77 (61.6)	48 (67.6)	22 (62.9)	23 (57.5)	20 (48.8)	324 (62.2)
Antibiotic intake (previous 30 days)	14 (16.9)	41 (32.5)^b^	35 (28.0)^b^	6 (8.5)	10 (28.6)^b^	7 (17.5)	7 (17.1)	120 (23.0)
Hospitalization (previous 3 months)	6 (7.2)	4 (3.2)	4 (3.2)	0 (0.0)	0 (0.0)	5 (12.5)	1 (2.4)	20 (3.8)
Previous ENT surgery^c^	5 (6.0)	9 (7.1)	7 (5.6)	12 (16.9)	3 (8.6)	8 (20.0)	5 (12.2)	49 (9.4)

^a^Significant (p = 0.001) differences between groups

^b^Significantly (p<0.01) higher than other groups

^c^Significant (p<0.05) differences between groups. SP = *S*. *pneumoniae*; Hi = *H*. *influenzae*.

Table 3 shows previous history of otitis and clinical data from the present episodes. Most episodes (66.6%) occurred in patients with past history of otitis, with statistical differences between children attending day care centres and who did not (73.8% vs. 54.8%, p<0.001). First episodes were positively associated with age <24 months (OR = 9.59; 95%CI = 4.40, 20.90, p<0.001) and 24–60 months (OR = 2.96; 95%CI = 1.35, 6.50, p = 0.007) in the multivariate model performed (R^2^ = 0.309, p<0.001) (see S2 Table for univariate analysis). Among first episodes of otitis, *S*. *pneumoniae* (25.3%) was the most frequent and *H*. *influenzae* (13.8%) the least frequent etiological agent. In the multivariate analysis (R^2^ = 0.291, p<0.001), first episodes of otitis by *S*. *pneumoniae* were positively associated with age <24 months (OR = 7.83; 95%CI = 2.73, 22.40; p<0.001), 24–60 months (OR = 2.94; 95%CI = 1.00, 8.61; p = 0.049), serotype 3 (OR = 3.29; 95%CI = 1.21, 8.94; p = 0.020) and serotype 19A (OR = 5.82; 95%CI = 2.42, 13.96; p<0.001) (see S3 Table for univariate analysis). With respect to otitis by *H*. *influenzae*, the model (R^2^ = 0.251, p<0.001) showed a positive association with age <24 months (OR = 5.39; 95%CI = 2.83, 10.27; p<0.001), 24–60 months (OR = 3.07; 95%CI = 1.62, 5.83; p = 0.001), recurrences (OR = 3.54; 95%CI = 2.28, 5.49; p<0.001) and nasopharyngeal carriage of *H*. *influenzae* (OR = 4.10; 95%CI = 2.67, 6.28; p<0.001) (see S4 Table for univariate analysis).

**Table 3:** Previous history of otitis and clinical data of children included in the study distributed by isolates from middle ear fluid.

	SP	Hi	SP + Hi	*S*. *pyogenes*	*S*. *aureus*	Other	Negative culture	Total
n	83	126	125	71	35	40	41	521
First OM episode	44 (53.0)	24 (19.0)	32 (25.6)	31 (43.7)	12 (34.3)	17 (42.5)	14 (34.1)	174 (33.4)
Previous OM episodes	39 (47.0)	102 (81.0)	93 (74.4)	40 (56.3)	23 (65.7)	23 (57.5)	27 (65.9)	347 (66.6)
One previous episode^a^	5 (6.0)	26 (20.6)	20 (16.0)	14 (19.7)	6 (17.1)	5 (12.5)	10 (24.4)	86 (16.6)
2–3 previous episodes^a^	14 (16.9)	30 (23.8)	39 (31.2)	12 (16.9)	11 (31.4)	6 (15.0)	7 (17.1)	119 (22.8)
≥4 previous episodes^a^	12 (14.5)	39 (31.0)	19 (15.2)	9 (12.7)	4 (11.4)	8 (20.0)	4 (9.8)	95 (18.2)
Unilateral OM	83 (100)	123 (97.6)	124 (99.2)	69 (97.2)	34 (97.1)	40 (100)	40 (97.6)	513 (98.5)
Earache^b^	62 (74.7)	81 (64.3)	74 (59.2)	55 (77.5)	25 (71.4)	30 (75.0)	32 (78.0)	359 (68.9)
Purulent exudate	59 (71.1)	94 (74.6)	83 (66.4)	53 (74.6)	27 (77.1)	32 (80.0)	33 (80.5)	372 (71.4)
Fever (≥38°C)	33 (39.8)	40 (31.7)	39 (31.2)	24 (33.8)	12 (34.3)	11 (27.5)	15 (36.6)	174 (33.4)
Pharyngitis	45 (54.2)	50 (39.7)	61 (48.8)	33 (46.5)	14 (40.0)	20 (50.0)	19 (46.3)	242 (46.4)
Adenopathy	12 (14.5)	12 (9.5)	13 (10.4)	11 (15.5)	4 (11.4)	6 (15.0)	10 (24.4)	68 (13.1)

^a^ Among patients with known data (n = 474)

^b^Significant (p = 0.019) differences between groups; SP = *S*. *pneumoniae*; Hi = *H*. *influenzae*.

**Fig 1:**
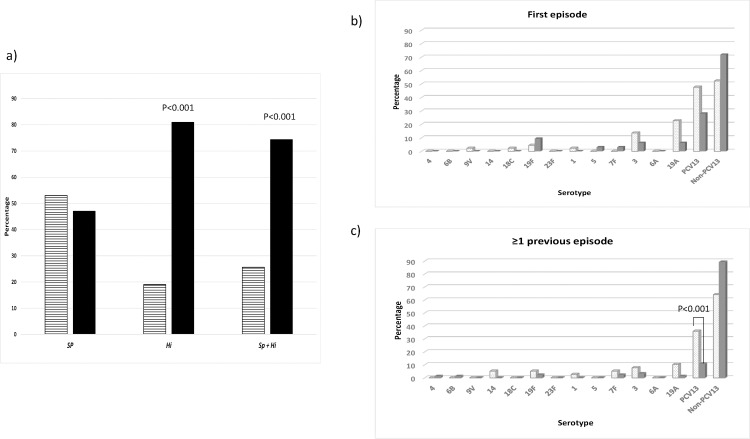
**Isolation rates of *S*. *pneumoniae* and/or *H*. *influenzae* in children with and without history of previous otitis episodes**: a) Percentage of children suffering a first episode of OM (striped columns) and with past history of otitis episodes (black columns) among children with OM by *S*. *pneumoniae*, *H*. *influenzae* and *S*. *pneumoniae* + *H*. *influenzae*; b) PCV13 serotypes in OM by *S*. *pneumoniae* (dotted columns) or *S*. *pneumoniae* + *H*. *influenzae* (grey columns) among children with a first episode of OM; c) Same as b) for children with past history of otitis.

Fig 1 shows differences in isolation rates (*S*. *pneumoniae*, *H*. *influenzae*) between first episodes and episodes from children with past history of otitis. Among OM involving *S*. *pneumoniae*, PCV13 serotypes were identified in 54 out of 208 (26.0%) cases. Fig 1 also shows involvement of PCV13 serotypes in OM by *S*. *pneumoniae* (whether or not concomitantly with *H*. *influenzae*) in first episodes of OM and in children with previous episodes. In mixed infections, the percentage of PCV13 serotypes was higher among first episodes [28.1% (9/32)] than among recurrences [10.8% (10/93)] (p = 0.0247). In the multivariate model (R^2^ = 0.132, p<0.001) otitis by PCV13 serotypes was positively associated with first episodes (OR = 2.23; 95%CI = 1.38, 3.60; p = 0.001) (see S5 Table for univariate analysis).

### Otopathogens carriage in nasopharynx

Carriage in nasopharynx of otopathogens was 42.0% for *S*. *pneumoniae*, 32.4% for *H*. *influenzae*, 13.2% for *S*. *pyogenes* and 3.1% for *S*. *aureus*. Table 4 shows the relationship of otopathogens in MEF and nasopharynx. Carriage in nasopharynx of species identified in MEF was found in 304/521 (58.3%) cases, with higher (p<0.001) carriage of the microorganism in nasopharynx when identified in MEF: 71.8% carriage of *S*. *pyogenes* among children with OM by this pathogen vs. 4.0% among children with other OM types, with percentages for *S*. *aureus* of 34.3% vs. 0.8%, for *H*. *influenzae* of 47.8% vs. 18.1%, and for *S*. *pneumoniae* of 58.2% vs. 31.0%.

**Table 4:** Isolation rates of otopathogens in middle ear fluid (MEF) and nasopharyngeal cultures.

	Nasopharyngeal swab												
MEF	*S*. *pneumoniae*		*H*. *influenzae*		*S*.*pneumoniae + H*. *influenzae*		*S*. *pyogenes*		*S*. *aureus*		Other		Total MEF
	n	%	n	%	n	%	n	%	n	%	n	%	n
***S*. *pneumoniae***	48	38.1	4	5.3	13	14.0	1	1.4	0	0.0	17	12.6	**83**
***H*. *influenzae***	17	13.5	37	48.7	32	34.4	4	5.8	2	12.5	32	23.7	**126**
***S*. *pneumoniae* + *H*. *influenzae***	28	22.2	19	25.0	32	34.4	7	10.1	0	0.0	37	27.4	**125**
***S*. *pyogenes***	9	7.1	1	1.3	1	1.1	51	73.9	1	6.3	8	5.9	**71**
***S*. *aureus***	8	6.3	5	6.6	1	1.1	1	1.4	12	75.0	7	5.2	**35**
**Others**	6	4.8	6	7.9	9	9.7	0	0.0	1	6.3	18	13.3	**40**
**Negative culture/PCR**	10	7.9	4	5.3	5	5.4	5	7.2	0	0.0	16	11.9	**41**
**Total Nasopharynx**	**126**		**76**		**93**		**69**		**16**		**135**		**521**^**a**^

^a^Six children had positive MEF sample and no nasopharynx sample.

In the multivariate analysis (R^2^ = 0.018, p = 0.012), carriage of *S*. *pneumoniae* and *H*. *influenzae* in nasopharynx was positively associated with otitis mixed infection (OR = 1.89; 95%CI = 1.16, 3.07; p = 0.010) (see S6 Table for univariate analysis).

### Pneumococcal serotypes in MEF and nasopharynx

**Fig 2:**
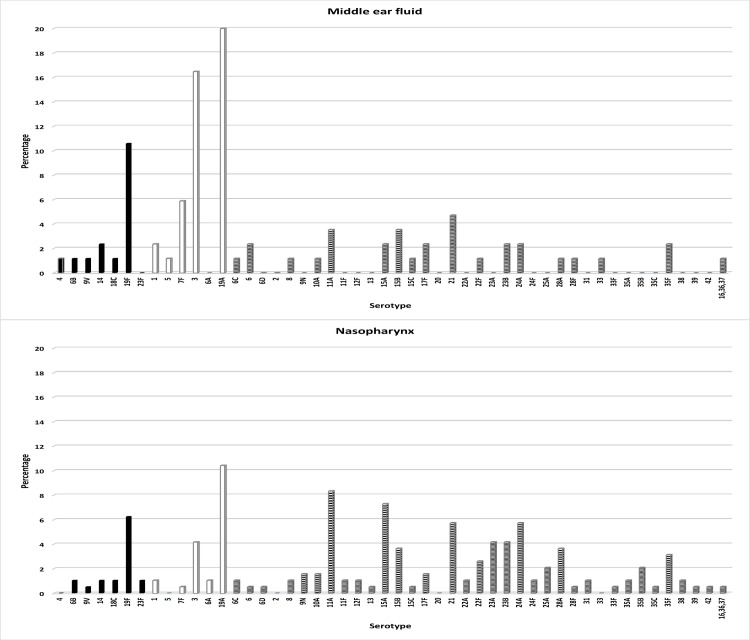
Serotypes identified in middle ear fluid and in nasopharynx among typed pneumococci (PCV7 serotypes: black columns; 6 newly added serotypes in PCV13: white columns; non-PCV13 serotypes: striped columns).

Fig 2 shows *S*. *pneumoniae* serotypes in MEF and nasopharynx among typed isolates. As shown in the figure, the distribution of serotypes in MEF and nasopharynx was different, with higher diversity in nasopharynx (higher presence of non-PCV13 serotypes) and higher involvement of PCV13 serotypes in MEF. The most frequent serotypes in MEF were serotypes 19A, 3, 19F and 7F, while in nasopharynx they were serotypes 19A, 11A, 15A, 19F, 21 and 24A.

Among the 121 OM involving *S*. *pneumoniae* with concomitant carriage of pneumococci in nasopharynx, the same serotype was identified in MEF and nasopharynx in 49 (40.5%) cases. Serotypes 19A and 3 showed the highest coincidences with 11 out of 17 (64.7%) and 6 out of 14 (42.9%) otitis by these serotypes, respectively.

### PCV13 vaccination, aetiology of OM, and carriage

The percentage of children having received ≥1 dose of one PCV was significantly higher among children attending day care centres than among those cared for at home (80.2% vs. 65.5%, p<0.001). Table 5 shows data on previous pneumococcal vaccination. Although 73.9% episodes occurred in children with history of pneumococcal vaccination, only 39.3% had received 3–4 doses of PCV13. The percentage of children with OM involving *S*. *pneumoniae* (with or without *H*. *influenzae*) was similar among non-vaccinated children and those who had received 3–4 doses of PCV13 (41.2% vs. 43.4%).

**Table 5:** Data on previous pneumococcal vaccination of children included in the study distributed by isolates from middle ear fluid.

	SP	Hi	SP + Hi	*S*. *pyogenes*	*S*. *aureus*	Other	Negative culture/PCR	Total
n	83	126	125	71	35	40	41	521
Previous pneumococcal vaccination	58 (69.9)	90 (71.4)	94 (75.2)	58 (81.7)	25 (71.4)	29 (72.5)	31 (75.6)	385 (73.9)
PCV7	10 (12.0)	13 (10.3)	12 (9.6)	16 (22.5)	6 (17.1)	13 (32.5)	9 (22.0)	79 (15.2)
PCV10	3 (3.6)	3 (2.4)	1 (0.8)	1 (1.4)	0 (0.0)	0 (0.0)	1 (2.4)	9 (1.7)
PCV13 (at least one dose)	45 (54.2)	74 (58.7)	81 (64.8)	41 (57.7)	19 (54.3)	16 (40.0)	21 (51.2)	297 (57.0)
PCV13 (3–4 doses)	30 (36.1)	52 (41.3)	59 (47.2)	29 (40.8)	8 (22.9)	11 (27.5)	16 (39.0)	205 (39.3)

SP = *S*. *pneumoniae*; Hi = *H*. *influenzae*; PCV = Pneumococcal conjugate vaccine.

With respect to pneumococcal carriage, 56 out of 136 (41.2%) non-vaccinated children and 129 out of 385 (33.5%) children who had received at least one PCV dose carried *S*. *pneumoniae* in nasopharynx, the difference being non-significant (p = 0.107). When carriage was compared in fully vaccinated (3–4 doses of PCV13) and in non-fully/non-vaccinated children, the difference did not reach significance [41/205 (20.0%) vs. 85/310 (27.4%), p = 0.059)]. For other otopathogens, no differences were found except for *S*. *pyogenes* with greater isolation in fully vaccinated children [36/205 (17.6%) vs. 33/310 (10.6%), p = 0.034)]. Carriage of PCV13 serotypes was more frequent among non-vaccinated children than among those who had received ≥1 dose of PCV13 (23/136 = 16.9% vs. 23/297 = 7.7%, p = 0.004), regardless of day care attendance.

The most frequent serotypes carried by non-vaccinated children were 19A (14.3%), 19F (10.7%), and serotypes 15A, 23B and 28A (5.4% each), while among vaccinated children (≥3 doses) the most frequent were serotypes 11A and 15A (11.3% each), 19A (8.8%), 21 (6.3%) and serotypes 19F, 23A and 24A (5.0% each).

### Antibiotic susceptibility in *S*. *pneumoniae* and *H*. *influenzae*

Among 280 *S*. *pneumoniae* culture isolates, percentages of non-susceptibility were 6.5% to penicillin, 5.4% to cefotaxime, 36.5% to erythromycin (52.2% *erm*B, 29.9% *erm*B/*mef*E, and 11.9% *mef*E among determined genotypes). Up to 30.0% isolates were multidrug resistant, with serotypes 19A (29.8%), 24A (14.3%), 19F (8.3%) and 15A (6.0%) including the highest percentage of multidrug resistant isolates.

Among 264 *H*. *influenzae* isolates tested, 3.8% were β-lactamase producing. Overall, non-susceptibility was 6.4% to ampicillin and 1.9% to amoxicillin/clavulanic acid.

### Antibiotic treatment

Up to 98.7% episodes were treated with antibiotics, without differences between groups, the most frequent compound used being amoxicillin/clavulanic acid (49.6%) and amoxicillin (32.7%). Children with past history of otitis were more frequently referred for ENT consultation (22.2% vs. 1.1%, p<0.001); of the 79 children referred, 50 (63.3%) presented OM involving *H*. *influenzae*.

## Discussion

This study, a large multicentre prospective study on OM and nasopharyngeal carriage in a Spanish region without PCVs included in the regional immunization programme, showed a significant decrease in the overall IR of OM over the study period, with significant reductions in IRs of OM by *S*. *pneumoniae* and by *H*. *influenzae*, but not in IRs of mixed OM by *S*. *pneumoniae* + *H*. *influenzae*, which represented a high proportion of OM. *H*. *influenzae* was the most frequent otopathogen, involved as single or mixed agent (with *S*. *pneumoniae*) in almost half cases of OM. It has been suggested that the decline of PCV13 serotypes after vaccine implementation may result in an increased relative predominance of *H*. *influenzae* as otopathogen [16,24]. Since in Catalonia, the Spanish region where our study was performed, a marked 72% reduction in vaccine-type otitis has been reported [25], the predominance of *H*. *influenzae* was not surprising. In addition, other factors associated in the literature with high *H*. *influenzae* involvement such as recurrences [26,27], day care attendance, PCV vaccination [16] or *H*. *influenzae* colonization at onset of infection [16] were also present in a high number of children in our series. In the multivariate model, OM by *H*. *influenzae* was associated with recurrences and nasopharyngeal colonization.

As in other studies [28,29], around a quarter of episodes in the present study were due to mixed *S*. *pneumoniae* + *H*. *influenzae* infections, which may be considered a different clinical entity [26]. This type of otitis is favoured by co-colonization, as indicated by our multivariate analysis and the literature [30,31] mainly for less virulent pneumococcal serotypes (33F, 6B, 15B, 10A and 11A) more associated with colonization than with invasive disease [32]. The IR of this entity showed small variations over the 3-year study period, with the highest IR in the last study period after the decline of IRs of OM by *H*. *influenzae* and by *S*. *pneumoniae* as single agents.

A special mention merits the high number of OM in our study that were etiologically diagnosed by the use of PCR, increasing around one-third the percentage of etiological culture-based diagnoses for OM involving *S*. *pneumoniae* or *H*. *influenzae* as single agents, a fact that was previously shown in pleural and cerebrospinal fluids samples [33]. In a previous study in otitis, the use of PCR highly increased identification rates not only for *S*. *pneumoniae* (from <10% to 23.1%) and *H*. *influenzae* (from 19.4% to 43.4%), but also for *M*. *catarrhalis* (from <10% to 38.7%) [27]. The use of PCR for detection of *M*. *catarrhalis* in our study would have increased the detection of this otopathogen with highly variable reported implication in otitis (from 0.8% [34] to 38.7% [27]) depending on study site and identification tools used. More importantly, in the present study, nearly 90% of *H*. *influenzae* + *S*. *pneumoniae* mixed OM were diagnosed by PCR, making PCR an essential tool for diagnosis due to the described interactions between these two species [35–38].

The pathogenesis of otitis involves recent (<2 weeks) colonization by a potential otopathogen [39,40], a fact that can be prevented in the case of *S*. *pneumoniae* trough vaccination. It has been postulated that nasopharyngeal cultures taken within one month [41] or at onset of acute otitis media [5] correlate better with aetiology, with percentages ranging from 68% to 97% [5]. The MEF-nasopharynx concordance found in the present study (≈58%) was in agreement with published data, and statistically significant for all otopathogens, with maximal rates in the case of *S*. *pyogenes* (around 70%) because of the lower carriage of *S*. *pneumoniae* and *H*. *influenzae* among children with OM by *S*. *pyogenes*. Although a negative association between *S*. *pneumoniae* and *S*. *aureus* has been reported [9,16], we could not find data on an association between *S*. *pneumoniae* and *S*. *pyogenes* to support our finding.

Pneumococcal nasopharyngeal colonization has profoundly changed in the post-vaccination era since vaccine serotypes have been highly reduced or have nearly disappeared [10,14,42,43], with subsequent reduction in otitis [13,15,39,44]. After PCV13 introduction, otitis media may decrease not only by direct PCV13 effectiveness in reducing vaccine-type otitis, but also as pneumococcal serotypes with greater capacity to cause disease are replaced by less pathogenic serotypes [45]. In the present study, pneumococcal carriage (as well as PCV13 carriage) was more frequent among non-vaccinated children. PCV13 serotypes (most of them classically considered as virulent serotypes) were more frequent in MEF than in nasopharynx (with high serotype diversity), and associated with first episodes in the multivariate analysis.

Two of these PCV13 serotypes, serotypes 19A and 3, were the most frequent pneumococcal serotypes causing OM in our series, both showing the highest rate of MEF-nasopharynx concordance. Serotype 19A greatly increased after PCV7 introduction, but rapidly declined after PCV13 implementation, in contrast to serotype 3, with reports of non-significant decreases following PCVs [15,43]. Serotype 19A was also identified as one of the most frequent serotypes carried by children who had received 3–4 doses of PCV13, as previously reported [43]. All non-PCV13 serotypes separately accounted for <5% in MEF, suggesting a lack of serotype replacement by non-vaccine serotypes.

It is noteworthy the percentage of ≈74% children with history of pneumococcal vaccination in our study. This percentage could not be assumed to reflect the situation in the total population in this area, where the estimated uptake is 50% [46]. Probably the fact that most of our children attended day care centres and had had previously more than one otitis episode contributed to this high vaccination rate. It has been postulated that only in communities where PCV13 coverage in children is ≥75% is there a decline in carriage of PCV13 serotypes in ≥50% of non-immunized children [47]. Considering the above stated particular reasons for our high vaccination rate, no herd effect among children <2 years should be suspected in our area. Increasing PCV13 vaccination would further reduce transmission of pneumococcal vaccine serotypes with special benefits for youngest children (with none or uncompleted vaccine schedules), preventing first episodes of OM and subsequent recurrent episodes (generally involving *H*. *influenzae* as single agent or as co-pathogen with *S*. *pneumoniae*).

An additional benefit to the decrease in PCV13 serotypes causing disease would be the decrease in the percentage of non-susceptibility to oral β-lactams. In this regard, it has been suggested that if after PCV13 implementation, serotype 19A (the major serotype showing multidrug resistance) continues to decline, decreasing levels of penicillin resistance would be expected perhaps to the point that lowering standard amoxicillin dose should be considered [43]. However, caution should be taken at least in our area with the replacement in the clonal composition of serotype 19A strains, with a significant increase in the Barcelona area of clone ST320 (with reduced susceptibility to oral β-lactam antibiotics and resistance to macrolides), confirming maintenance of high amoxicillin doses or ceftriaxone as the current recommendation [48]. Interestingly, the percentage of phenotypically resistant *H*. *influenzae* isolates in the present study was low, but ≈50% children treated with antibiotics received amoxicillin/clavulanic acid. A previous report has suggested that although *H*. *influenzae* is currently the most frequent otopathogen, due to the decreasing proportion of β-lactam-resistant *H*. *influenzae* isolates, the prescription of amoxicillin/clavulanate as first line seems to have no benefit or justification [49].

Several limitations of the present study may be identified. First, the study was focused on children with otitis presenting spontaneous tympanic perforation and isolates may not be representative of the whole spectrum of otitis. Second, the percentage of children having received ≥1 dose of PCVs was higher than that reported in our geographical area and in Spain at the study time, indicating special characteristics of children (day care attendance, previous recurrences, etc.) in the present study, not allowing conclusions on herd effect. Third, only serotypes 1, 3, 4, 5, 6, 7F, 14, 19A and 19F were identified by the real-time PCR assay used for pneumococci identified by PCR, and genotyping was not performed. Finally, PCR was only performed in samples showing negative culture, not addressing the possibility of positive PCR for *S*. *pneumoniae* in samples yielding positive culture to *H*. *influenzae* and *vice versa*. However, the multicentre design of the study including children attended as primary care, the large number of children included over 3 years, and the PCR detection of *S*. *pneumoniae* and *H*. *influenzae* in culture negative samples represent important strengths of the study.

## Conclusions

This study, a large multicentre prospective study on OM and nasopharyngeal carriage in a Spanish region without PCVs in the regional immunization programme, showed >50% MEF-nasopharyngeal concordance of otopathogen isolation, differences in aetiology between first episodes (associated with *S*. *pneumoniae* infection) and recurrent episodes (associated with *H*. *influenzae* involvement), and association of PCV13 serotypes with first episodes. Our results suggest that increasing PCV13 vaccination would further reduce transmission of pneumococcal vaccine serotypes with special benefits for youngest children (with none or uncompleted vaccine schedules), preventing first episodes of OM and subsequent recurrent episodes.

## Supporting Information

S1 Table Univariate analysis for OM by *S*. *pneumonia*. (DOCX)

S2 Table Univariate analysis for first episode of OM. (DOCX)

S3 Table Univariate analysis for first OM episode by *S*. *pneumonia*. (DOCX)

S4 Table Univariate analysis for OM by *H*. *influenza*. (DOCX)

S5 Table Univariate analysis for OM by PCV13 serotypes. (DOCX)

S6 Table Univariate analysis for OM caused by *S*. *pneumoniae* + *H*. *influenza*. (DOCX)
