# Supplementary material for: Epidemiology of Otitis Media with Spontaneous Perforation of the Tympanic Membrane in Young Children and Association with Bacterial Nasopharyngeal Carriage, Recurrences and Pneumococcal Vaccination in Catalonia, Spain - The Prospective HERMES Study
Source: PLoS One. 2017 Feb 1;12(2):e0170316. doi: 10.1371/journal.pone.0170316 (PMC5287464; doi:10.1371/journal.pone.0170316)
Supplement: S1 Table — (DOCX) [file pone.0170316.s001.docx]

**Table S1.** Univariate analysis for OM by *S. pneumoniae*

|  | **Total (n=521)** | | **NO**  **(n=313)** | | **YES**  **(n=208)** | | **B** | **p** | **OR** | **95%CI** | |
| --- | --- | --- | --- | --- | --- | --- | --- | --- | --- | --- | --- |
|  | **n** | **%** | **n** | **%** | **n** | **%** |  |  |  | **Lower** | **Upper** |
| - **>60 months** | 72 | 13.8 | 52 | 16.6 | 20 | 9.6 |  | 0.028 |  |  |  |
| - **< 24 months** | 259 | 49.7 | 143 | 45.7 | 116 | 55.8 | 0.746 | 0.010 | 2.109 | 1.192 | 3.733 |
| - **24-60 months** | 190 | 36.5 | 118 | 37.7 | 72 | 34.6 | 0.461 | 0.127 | 1.586 | 0.877 | 2.871 |
| **Premature** | 36 | 6.9 | 20 | 6.4 | 16 | 7.7 | 0.200 | 0.566 | 1.221 | 0.617 | 2.415 |
| **Common cold (previous 15 days)** | 338 | 64.9 | 192 | 61.3 | 146 | 70.2 | 0.395 | 0.039 | 1.484 | 1.021 | 2.158 |
| **Day care attendance** | 324 | 62.2 | 199 | 63.6 | 125 | 60.1 | -0.148 | 0.422 | 0.863 | 0.602 | 1.237 |
| **Hospitalization (previous 3 months)** | 20 | 3.8 | 10 | 3.2 | 10 | 4.8 | 0.425 | 0.351 | 1.530 | 0.626 | 3.744 |
| **Antibiotic treatment (previous 30 days)** | 120 | 23.6 | 71 | 23.3 | 49 | 24.1 | 0.048 | 0.823 | 1.049 | 0.691 | 1.591 |
| **Previous OM episodes** | 347 | 66.6 | 215 | 68.7 | 132 | 63.5 | -0.234 | 0.216 | 0.792 | 0.547 | 1.146 |
| **No pneumococcal vaccination** | 136 | 26.1 | 80 | 25.6 | 56 | 26.9 |  | 0.132 |  |  |  |
| - **At least one PCV7 dose** | 79 | 15.2 | 57 | 18.2 | 22 | 10.6 | -0.595 | 0.051 | 0.551 | 0.303 | 1.004 |
| - **At least one PCV10 dose** | 9 | 1.7 | 5 | 1.6 | 4 | 1.9 | 0.134 | 0.847 | 1.143 | 0.294 | 4.446 |
| - **At least one PCV13 dose** | 297 | 57.0 | 171 | 54.6 | 126 | 60.6 | 0.051 | 0.807 | 1.053 | 0.697 | 1.589 |
| ***S. pneumoniae* in nasopharynx** | 169 | 32.4 | 101 | 32.3 | 68 | 32.7 | 0.019 | 0.919 | 1.020 | 0.701 | 1.482 |
