# Supplementary material for: Epidemiology of Otitis Media with Spontaneous Perforation of the Tympanic Membrane in Young Children and Association with Bacterial Nasopharyngeal Carriage, Recurrences and Pneumococcal Vaccination in Catalonia, Spain - The Prospective HERMES Study
Source: PLoS One. 2017 Feb 1;12(2):e0170316. doi: 10.1371/journal.pone.0170316 (PMC5287464; doi:10.1371/journal.pone.0170316)
Supplement: S2 Table — (DOCX) [file pone.0170316.s002.docx]

**Table S2.** Univariate analysis for first episode of OM

|  | **Total (n=521)** | | **NO**  **(n=347)** | | **YES**  **(n=174)** | | **B** | **p** | **OR** | **95%CI** | |
| --- | --- | --- | --- | --- | --- | --- | --- | --- | --- | --- | --- |
|  | **n** | **%** | **n** | **%** | **n** | **%** |  |  |  | **Lower** | **Upper** |
| - **>60 months** | 72 | 13.8 | 62 | 17.9 | 10 | 5.7 |  | 0.000 |  |  |  |
| - **< 24 months** | 259 | 49.7 | 146 | 42.1 | 113 | 64.9 | 1.568 | 0.000 | 4.799 | 2.355 | 9.776 |
| - **24-60 months** | 190 | 36.5 | 139 | 40.1 | 51 | 29.3 | 0.822 | 0.030 | 2.275 | 1.084 | 4.773 |
| **Premature** | 36 | 6.9 | 28 | 8.1 | 8 | 4.6 | -0.600 | 0.146 | 0.549 | 0.245 | 1.232 |
| **Common cold (previous 15 days)** | 338 | 64.9 | 218 | 62.8 | 120 | 69.0 | 0.274 | 0.167 | 1.315 | 0.892 | 1.938 |
| **Day care attendance** | 324 | 62.2 | 239 | 68.9 | 85 | 48.9 | -0.840 | 0.000 | 0.432 | 0.297 | 0.627 |
| **Hospitalization (previous 3 months)** | 20 | 3.8 | 10 | 2.9 | 10 | 5.7 | 0.720 | 0.115 | 2.055 | 0.839 | 5.035 |
| **Antibiotic treatment (previous 30 days)** | 120 | 23.6 | 107 | 31.5 | 13 | 7.7 | -1.700 | 0.000 | 0.183 | 0.099 | 0.336 |
| **No pneumococcal vaccination** | 136 | 26.1 | 79 | 22.8 | 57 | 32.8 |  | 0.027 |  |  |  |
| - **At least one PCV7 dose** | 79 | 15.2 | 60 | 17.3 | 19 | 10.9 | -0.824 | 0.009 | 0.439 | 0.237 | 0.814 |
| - **At least one PCV10 dose** | 9 | 1.7 | 8 | 2.3 | 1 | .6 | -1.753 | 0.103 | 0.173 | 0.021 | 1.424 |
| - **At least one PCV13 dose** | 297 | 57.0 | 200 | 57.6 | 97 | 55.7 | -0.397 | 0.063 | 0.672 | 0.442 | 1.021 |
| **Isolate from MEF** |  |  |  |  |  |  |  |  |  |  |  |
| - ***S. pneumoniae*** | 83 | 15.9 | 39 | 11.2 | 44 | 25.3 | 0.544 | 0.041 | 1.723 | 1.023 | 2.901 |
| - ***H. influenzae*** | 126 | 24.2 | 102 | 29.4 | 24 | 13.8 | -1.024 | 0.000 | 0.359 | 0.211 | 0.612 |
| - ***S. pneumoniae + H. influenzae*** | 125 | 24.0 | 93 | 26.8 | 32 | 18.4 | -0.644 | 0.011 | 0.525 | 0.320 | 0.864 |
| - **Others** | 187 | 35.9 | 113 | 32.6 | 74 | 42.5 |  | 0.000 |  |  |  |
| **Isolate from nasopharynx** |  |  |  |  |  |  |  |  |  |  |  |
| - ***S. pneumoniae*** | 126 | 24.2 | 73 | 21.0 | 53 | 30.5 | 0.400 | 0.082 | 1.491 | 0.951 | 2.338 |
| - ***H. influenzae*** | 76 | 14.6 | 49 | 14.1 | 27 | 15.5 | 0.124 | 0.657 | 1.132 | 0.656 | 1.953 |
| - ***S. pneumoniae + H. influenzae*** | 93 | 17.9 | 73 | 21.0 | 20 | 11.5 | -0.575 | 0.047 | 0.563 | 0.319 | 0.992 |
| - **Others** | 226 | 43.4 | 152 | 43.8 | 74 | 42.5 |  | 0.018 |  |  |  |
