# Supplementary material for: Epidemiology of Otitis Media with Spontaneous Perforation of the Tympanic Membrane in Young Children and Association with Bacterial Nasopharyngeal Carriage, Recurrences and Pneumococcal Vaccination in Catalonia, Spain - The Prospective HERMES Study
Source: PLoS One. 2017 Feb 1;12(2):e0170316. doi: 10.1371/journal.pone.0170316 (PMC5287464; doi:10.1371/journal.pone.0170316)
Supplement: S3 Table — (DOCX) [file pone.0170316.s003.docx]

**Table S3.** Univariate analysis for first OM episode by *S. pneumoniae*

|  | **Total**  **(n=427)** | | **NO**  **(n=278)** | | **YES**  **(n=149)** | | **B** | **p** | **OR** | **95%CI** | |
| --- | --- | --- | --- | --- | --- | --- | --- | --- | --- | --- | --- |
|  | **n** | **%** | **n** | **%** | **n** | **%** |  |  |  | **Lower** | **Upper** |
| - **> 60 months** | 37 | 8.7 | 32 | 11.5 | 5 | 3.4 |  | 0.000 |  |  |  |
| - **< 24 months** | 231 | 54.1 | 130 | 46.8 | 101 | 67.8 | 1.604 | 0.001 | 4.972 | 1.870 | 13.218 |
| - **24-60 months** | 159 | 37.2 | 116 | 41.7 | 43 | 28.9 | 0.864 | 0.092 | 2.372 | 0.868 | 6.484 |
| **Premature** | 32 | 7.5 | 22 | 7.9 | 10 | 6.7 | -0.178 | 0.653 | .837 | 0.385 | 1.818 |
| **Common cold (previous 15 days)** | 292 | 68.4 | 190 | 68.3 | 102 | 68.5 | 0.005 | 0.981 | 1.005 | 0.655 | 1.542 |
| **Day care attendance** | 257 | 60.2 | 189 | 68.0 | 68 | 45.6 | -0.928 | 0.000 | 0.395 | 0.263 | 0.595 |
| **Hospitalization (previous 3 months)** | 23 | 5.4 | 13 | 4.7 | 10 | 6.7 | 0.383 | 0.377 | 1.467 | 0.627 | 3.430 |
| **Antibiotic treatment (previous 30 days)** | 93 | 22.4 | 82 | 30.3 | 11 | 7.6 | -1.665 | 0.000 | 0.189 | 0.097 | 0.369 |
| **PCV13 serotypes** | 108 | 25.3 | 53 | 19.1 | 55 | 36.9 | 0.910 | 0.000 | 2.484 | 1.588 | 3.886 |
| **Serotype 4** | 1 | 0.2 | 1 | 0.4 | 0 | 0.0 | -20.583 | 1.000 | - | - | - |
| **Serotype 6B** | 3 | 0.7 | 3 | 1.1 | 0 | 0.0 | -20.590 | 0.999 | - | - | - |
| **Serotype 9V** | 2 | 0.5 | 0 | 0.0 | 2 | 1.3 | 21.840 | 0.999 | - | - | - |
| **Serotype 14** | 4 | 0.9 | 4 | 1.4 | 0 | 0.0 | -20.594 | 0.999 | - | - | - |
| **Serotype 18C** | 3 | 0.7 | 1 | 0.4 | 2 | 1.3 | 1.327 | 0.280 | 3.769 | 0.339 | 41.909 |
| **Serotype 19F** | 21 | 4.9 | 10 | 3.6 | 11 | 7.4 | 0.759 | 0.091 | 2.136 | 0.885 | 5.154 |
| **Serotype 23F** | 2 | 0.5 | 1 | 0.4 | 1 | 0.7 | 0.627 | 0.658 | 1.872 | 0.116 | 30.138 |
| **Serotype 1** | 4 | 0.9 | 2 | 0.7 | 2 | 1.3 | 0.630 | 0.531 | 1.878 | 0.262 | 13.465 |
| **Serotype 5** | 1 | 0.2 | 0 | 0.0 | 1 | 0.7 | 21.833 | 1.000 | - | - | - |
| **Serotype 7F** | 6 | 1.4 | 5 | 1.8 | 1 | 0.7 | -0.997 | 0.365 | 0.369 | 0.043 | 3.187 |
| **Serotype 3** | 22 | 5.2 | 10 | 3.6 | 12 | 8.1 | 0.853 | 0.053 | 2.347 | 0.989 | 5.570 |
| **Serotype 6A** | 2 | 0.5 | 2 | 0.7 | 0 | 0.0 | -20.586 | 0.999 | - | - | - |
| **Serotype 19A** | 37 | 8.7 | 14 | 5.0 | 23 | 15.4 | 1.236 | 0.001 | 3.442 | 1.714 | 6.914 |
