# Supplementary material for: Epidemiology of Otitis Media with Spontaneous Perforation of the Tympanic Membrane in Young Children and Association with Bacterial Nasopharyngeal Carriage, Recurrences and Pneumococcal Vaccination in Catalonia, Spain - The Prospective HERMES Study
Source: PLoS One. 2017 Feb 1;12(2):e0170316. doi: 10.1371/journal.pone.0170316 (PMC5287464; doi:10.1371/journal.pone.0170316)
Supplement: S4 Table — (DOCX) [file pone.0170316.s004.docx]

**Table S4.** Univariate analysis for OM by *H. influenzae*

|  | **Total (N=521)** | | **NO**  **(N=270)** | | **YES**  **(N=251)** | | **B** | **p** | **OR** | **95%CI** | |
| --- | --- | --- | --- | --- | --- | --- | --- | --- | --- | --- | --- |
|  | **N** | **%** | **N** | **%** | **N** | **%** |  |  |  | **Lower** | **Upper** |
| - **>60 months** | 72 | 13.8 | 53 | 19.6 | 19 | 7.6 |  | 0.000 |  |  |  |
| - **< 24 months** | 259 | 49.7 | 115 | 42.6 | 144 | 57.4 | 1.251 | 0.000 | 3.493 | 1.958 | 6.230 |
| - **24-60 months** | 190 | 36.5 | 102 | 37.8 | 88 | 35.1 | 0.878 | 0.004 | 2.407 | 1.325 | 4.370 |
| **Premature** | 36 | 6.9 | 23 | 8.5 | 13 | 5.2 | -0.533 | 0.137 | 0.587 | 0.290 | 1.185 |
| **Common cold (previous 15 days)** | 338 | 64.9 | 174 | 64.4 | 164 | 65.3 | 0.039 | 0.831 | 1.040 | 0.726 | 1.491 |
| **Day care attendance** | 324 | 62.2 | 161 | 59.6 | 163 | 64.9 | 0.226 | 0.212 | 1.254 | 0.879 | 1.789 |
| **Hospitalization (previous 3 months)** | 20 | 3.8 | 12 | 4.4 | 8 | 3.2 | -0.346 | 0.457 | 0.708 | 0.284 | 1.761 |
| **Antibiotic treatment (previous 30 days)** | 120 | 23.6 | 44 | 16.9 | 76 | 30.6 | 0.774 | 0.000 | 2.169 | 1.423 | 3.307 |
| **Previous OM episodes** | 347 | 66.6 | 152 | 56.3 | 195 | 77.7 | 0.994 | 0.000 | 2.703 | 1.844 | 3.962 |
| **No pneumococcal vaccination** | 136 | 26.1 | 69 | 25.6 | 67 | 26.7 |  | 0.016 |  |  |  |
| - **At least one PCV7 dose** | 79 | 15.2 | 54 | 20.0 | 25 | 10.0 | -0.741 | 0.012 | 0.477 | 0.267 | 0.853 |
| - **At least one PCV10 dose** | 9 | 1.7 | 5 | 1.9 | 4 | 1.6 | -0.194 | 0.780 | 0.824 | 0.212 | 3.201 |
| - **At least one PCV13 dose** | 297 | 57.0 | 142 | 52.6 | 155 | 61.8 | 0.117 | 0.572 | 1.124 | 0.749 | 1.687 |
| ***H. influenzae* in nasopharynx** | 169 | 32.4 | 49 | 18.1 | 120 | 47.8 | 1.419 | 0.000 | 4.131 | 2.779 | 6.141 |
