# Supplementary material for: Epidemiology of Otitis Media with Spontaneous Perforation of the Tympanic Membrane in Young Children and Association with Bacterial Nasopharyngeal Carriage, Recurrences and Pneumococcal Vaccination in Catalonia, Spain - The Prospective HERMES Study
Source: PLoS One. 2017 Feb 1;12(2):e0170316. doi: 10.1371/journal.pone.0170316 (PMC5287464; doi:10.1371/journal.pone.0170316)
Supplement: S5 Table — (DOCX) [file pone.0170316.s005.docx]

**Table S5.** Univariate analysis for OM by PCV13 serotypes

|  | **Total**  **(n=427)** | | **NO**  **(n=319)** | | **YES**  **(n=108)** | | **B** | **p** | **OR** | **95%CI** | |
| --- | --- | --- | --- | --- | --- | --- | --- | --- | --- | --- | --- |
|  | **n** | **%** | **n** | **%** | **n** | **%** |  |  |  | **Lower** | **Upper** |
| - **>60 months** | 37 | 8.7 | 27 | 8.5 | 10 | 9.3 |  | 0.870 |  |  |  |
| - **<24 months** | 231 | 54.1 | 171 | 53.6 | 60 | 55.6 | -0.054 | 0.892 | 0.947 | 0.433 | 2.073 |
| - **24-60 months** | 159 | 37.2 | 121 | 37.9 | 38 | 35.2 | -0.165 | 0.690 | 0.848 | 0.376 | 1.910 |
| **Premature** | 32 | 7.5 | 28 | 8.8 | 4 | 3.7 | -0.917 | 0.093 | 0.400 | 0.137 | 1.167 |
| **Common cold (previous 15 days)** | 292 | 68.4 | 220 | 69.0 | 72 | 66.7 | -0.105 | 0.657 | 0.900 | 0.565 | 1.433 |
| **Day care attendance** | 257 | 60.2 | 206 | 64.6 | 51 | 47.2 | -0.712 | 0.002 | 0.491 | 0.315 | 0.764 |
| **Hospitalization (previous 3 months)** | 23 | 5.4 | 18 | 5.6 | 5 | 4.6 | -0.209 | 0.687 | 0.812 | 0.294 | 2.242 |
| **Antibiotic treatment (previous 30 days)** | 93 | 22.4 | 72 | 23.2 | 21 | 19.8 | -0.203 | 0.467 | 0.817 | 0.473 | 1.409 |
| **Previous OM episodes** | 149 | 34.9 | 94 | 29.5 | 55 | 50.9 | 0.910 | 0.000 | 2.484 | 1.588 | 3.886 |
| **No pneumococcal vaccination** | 107 | 25.4 | 63 | 20.1 | 44 | 40.7 |  | 0.000 |  |  |  |
| - **At least one PCV7 dose** | 53 | 12.6 | 43 | 13.7 | 10 | 9.3 | -1.100 | 0.006 | 0.333 | 0.151 | 0.733 |
| - **At least one PCV10 dose** | 7 | 1.7 | 3 | 1.0 | 4 | 3.7 | 0.647 | 0.412 | 1.909 | 0.407 | 8.956 |
| - **At least one PCV13 dose** | 255 | 60.4 | 205 | 65.3 | 50 | 46.3 | -1.052 | 0.000 | 0.349 | 0.213 | 0.572 |
