# Supplementary material for: Epidemiology of Otitis Media with Spontaneous Perforation of the Tympanic Membrane in Young Children and Association with Bacterial Nasopharyngeal Carriage, Recurrences and Pneumococcal Vaccination in Catalonia, Spain - The Prospective HERMES Study
Source: PLoS One. 2017 Feb 1;12(2):e0170316. doi: 10.1371/journal.pone.0170316 (PMC5287464; doi:10.1371/journal.pone.0170316)
Supplement: S6 Table — (DOCX) [file pone.0170316.s006.docx]

**Table S6.** Univariate analysis for OM caused by *S. pneumoniae* + *H. influenzae*

|  | **Total (n=521)** | | **NO**  **(n=396)** | | **YES**  **(n=125)** | | **B** | **p** | **OR** | **95%CI** | |
| --- | --- | --- | --- | --- | --- | --- | --- | --- | --- | --- | --- |
|  | **n** | **%** | **n** | **%** | **n** | **%** |  |  |  | **Lower** | **Upper** |
| - **>60 months** | 72 | 13.8 | 61 | 15.4 | 11 | 8.8 |  | 0.093 |  |  |  |
| - **< 24 months** | 259 | 49.7 | 188 | 47.5 | 71 | 56.8 | 0.739 | 0.038 | 2.094 | 1.042 | 4.208 |
| - **24-60 months** | 190 | 36.5 | 147 | 37.1 | 43 | 34.4 | 0.484 | 0.192 | 1.622 | 0.785 | 3.354 |
| **Premature** | 36 | 6.9 | 29 | 7.3 | 7 | 5.6 | -0.287 | 0.509 | 0.751 | 0.321 | 1.758 |
| **Common cold (previous 15 days)** | 338 | 64.9 | 252 | 63.6 | 86 | 68.8 | 0.231 | 0.292 | 1.260 | 0.820 | 1.937 |
| **Day care attendance** | 324 | 62.2 | 247 | 62.4 | 77 | 61.6 | -0.033 | 0.876 | 0.968 | 0.640 | 1.464 |
| **Hospitalization (previous 3 months)** | 20 | 3.8 | 16 | 4.0 | 4 | 3.2 | -0.242 | 0.671 | 0.785 | 0.258 | 2.393 |
| **Antibiotic treatment (previous 30 days)** | 120 | 23.6 | 85 | 22.1 | 35 | 28.5 | 0.339 | 0.148 | 1.404 | 0.886 | 2.223 |
| **Previous OM episodes** | 347 | 66.6 | 254 | 64.1 | 93 | 74.4 | 0.485 | 0.035 | 1.625 | 1.035 | 2.551 |
| **No pneumococcal vaccination** | 136 | 26.1 | 105 | 26.5 | 31 | 24.8 |  | 0.119 |  |  |  |
| - **At least one PCV7 dose** | 79 | 15.2 | 67 | 16.9 | 12 | 9.6 | -0.500 | 0.182 | 0.607 | 0.291 | 1.263 |
| - **At least one PCV10 dose** | 9 | 1.7 | 8 | 2.0 | 1 | .8 | -0.859 | 0.426 | 0.423 | 0.051 | 3.517 |
| - **At least one PCV13 dose** | 297 | 57.0 | 216 | 54.5 | 81 | 64.8 | 0.239 | 0.324 | 1.270 | 0.790 | 2.043 |
| ***S. pneumoniae + H. influenzae*  in nasopharynx** | 93 | 17.9 | 61 | 15.4 | 32 | 25.6 | 0.636 | 0.010 | 1.890 | 1.163 | 3.071 |
